# Author Correction: Introducing Biomedisa as an open-source online platform for biomedical image segmentation

**DOI:** 10.1038/s41467-024-53245-x

**Published:** 2024-10-23

**Authors:** Philipp D. Lösel, Thomas van de Kamp, Alejandra Jayme, Alexey Ershov, Tomáš Faragó, Olaf Pichler, Nicholas Tan Jerome, Narendar Aadepu, Sabine Bremer, Suren A. Chilingaryan, Michael Heethoff, Andreas Kopmann, Janes Odar, Sebastian Schmelzle, Marcus Zuber, Joachim Wittbrodt, Tilo Baumbach, Vincent Heuveline

**Affiliations:** 1 Engineering Mathematics and Computing Lab (EMCL), Interdisciplinary Center for Scientific Computing (IWR), Heidelberg University, Im Neuenheimer Feld 205, 69120 Heidelberg, Germany; 2 Heidelberg Institute for Theoretical Studies (HITS), Schloss-Wolfsbrunnenweg 35, 69118 Heidelberg, Germany; 3 Institute for Photon Science and Synchrotron Radiation (IPS), Karlsruhe Institute of Technology (KIT), Hermann-von-Helmholtz-Platz 1, 76344 Eggenstein-Leopoldshafen, Germany; 4 Laboratory for Applications of Synchrotron Radiation (LAS), Karlsruhe Institute of Technology (KIT), Kaiserstr. 12, 76131 Karlsruhe, Germany; 5 Heidelberg University Computing Centre (URZ), Im Neuenheimer Feld 293, 69120 Heidelberg, Germany; 6 Institute for Data Processing and Electronics (IPE), Karlsruhe Institute of Technology (KIT), Hermann-von-Helmholtz-Platz 1, 76344 Eggenstein-Leopoldshafen, Germany; 7 Centre for Organismal Studies Heidelberg (COS), Heidelberg University, Im Neuenheimer Feld 230, 69120 Heidelberg, Germany; 8 Institute of Biological and Chemical Systems (IBCS), Karlsruhe Institute of Technology (KIT), Hermann-von-Helmholtz-Platz 1, 76344 Eggenstein-Leopoldshafen, Germany; 9 Ecological Networks, Technical University of Darmstadt, Schnittspahnstr. 3, 64287 Darmstadt, Germany

Correction to: *Nature Communications* 10.1038/s41467-020-19303-w, published online 4 November 2020

The original version of this Article contained a domain name for the Segmentation App described that has since been updated. This was written in the Introduction and Data Availability statement, which incorrectly read ‘https://biomedisa.org’. The correct version replaces this with ‘https://biomedisa.info’.

The original version of this Article gave the email address of the corresponding Author as philipp.loesel@uni-heidelberg.de. This has since been updated to philipp.david.loesel@gmail.com.

This has now been corrected in both the PDF and HTML versions of the Article.

