# Supplementary material for: Introducing Biomedisa as an open-source online platform for biomedical image segmentation
Source: Nat Commun. 2020 Nov 4;11:5577. doi: 10.1038/s41467-020-19303-w (PMC7642381; doi:10.1038/s41467-020-19303-w)
Supplement: Supplementary file 1 — Supplementary Information [file 41467_2020_19303_MOESM1_ESM.pdf]

Supplementary Information

Introducing Biomedisa as an open-source online platform for biomedical image segmentation

Lösel *et al.*

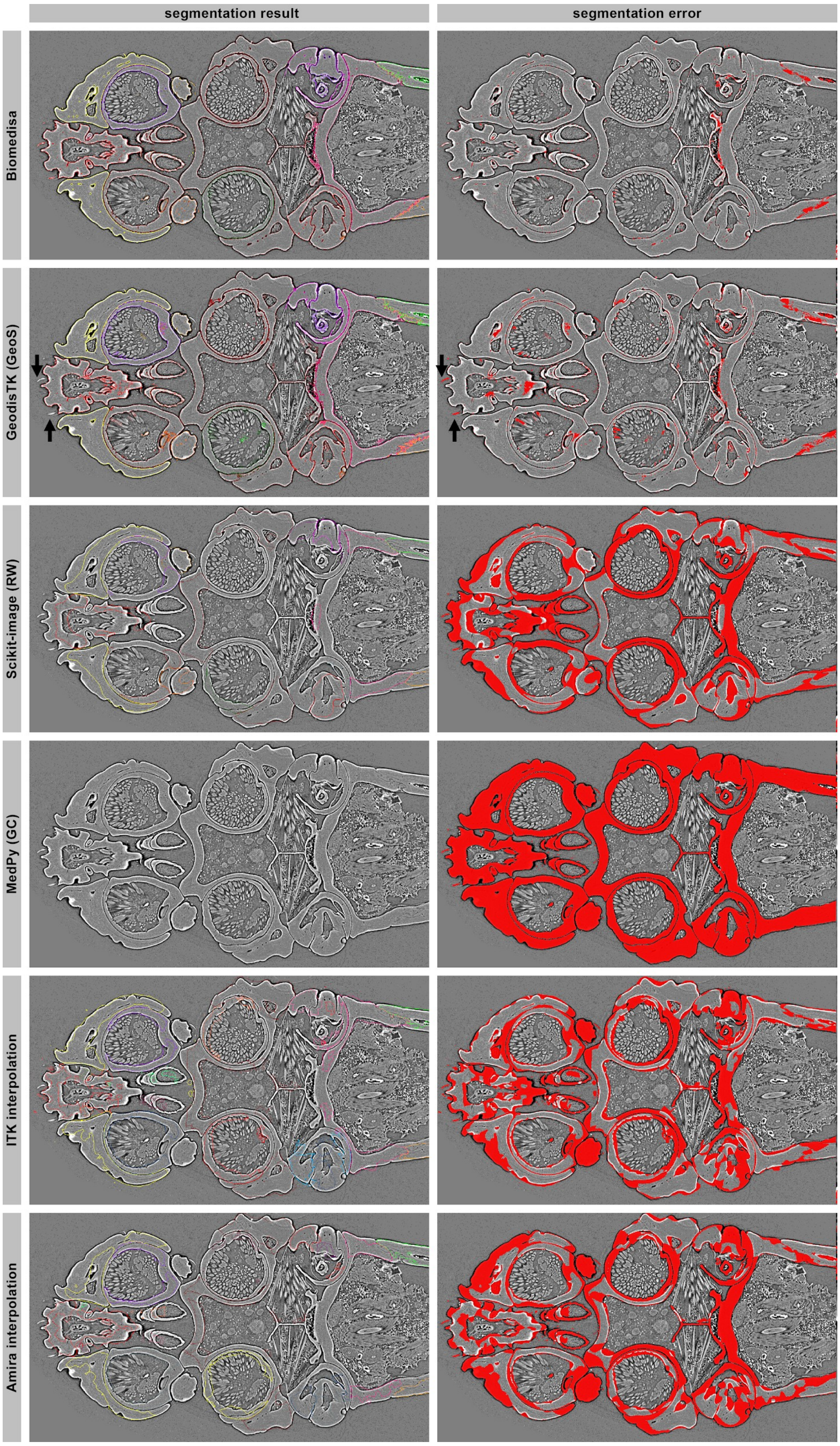

Supplementary Fig. 1 | Visual comparison of the segmentation results of different semi-automatic segmentation tools when segmenting a *Trigonopterus* weevil with a pre-segmentation that is adapted to the morphology of the weevil. The standard configuration is used for all techniques. The segmentation errors shown here are based on the manual ground truth data, in which every 5th slice was labeled by an expert. GeoS was unable to segment the beetle's tiny hair (arrows). Large body parts are missing in the segmentation results of RW, ITK and Amira. The segmentation of the section shown contains no body parts segmented by GC.

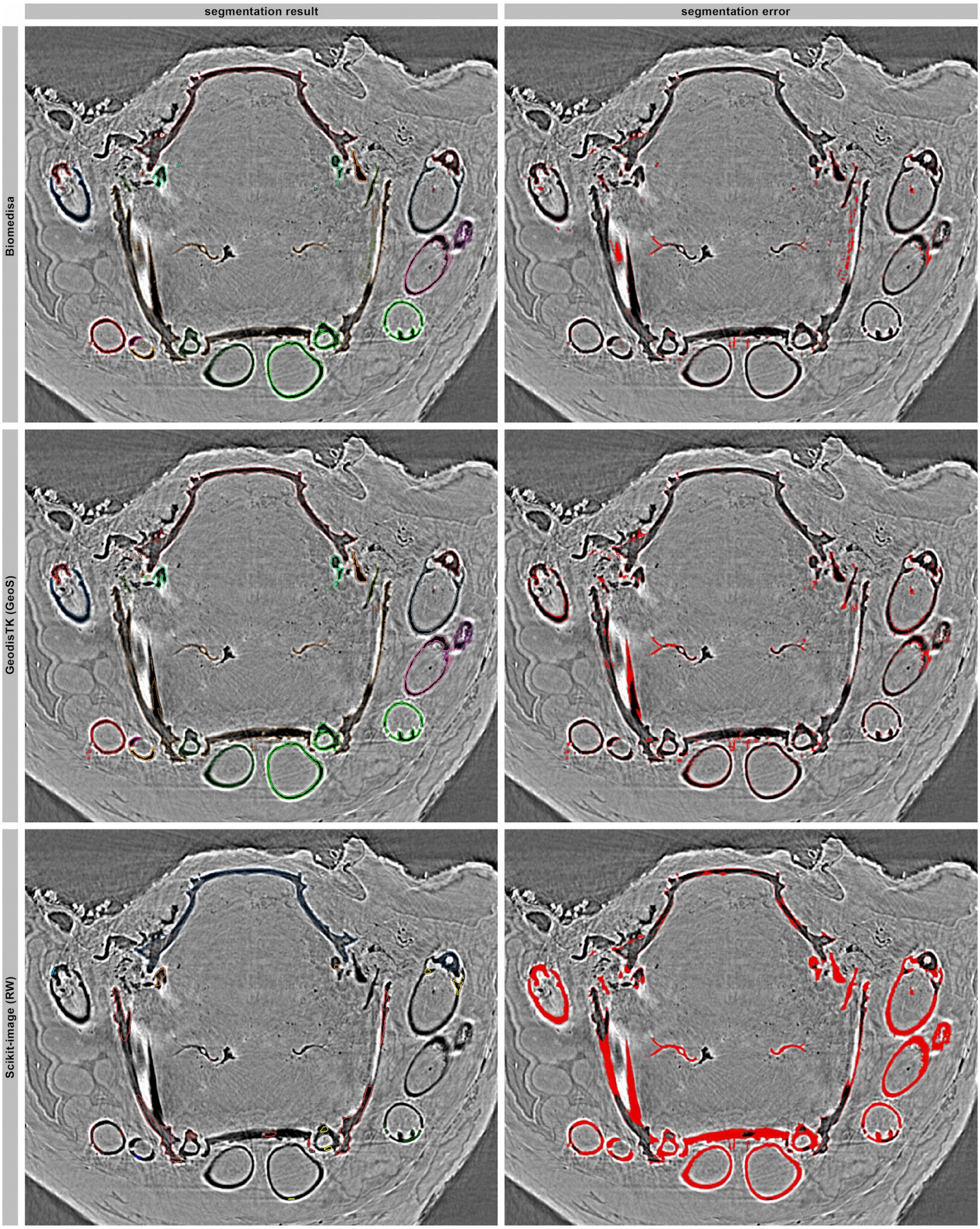

Supplementary Fig. 2 | Visual comparison of the segmentation results of different semi-automatic segmentation tools when segmenting a mineralized wasp. The standard configuration is used for all techniques. Half of the originally pre-segmented slices were used to obtain the segmentation results. The segmentation errors shown here are based on the remaining pre-segmented slices that have been labeled by an expert.

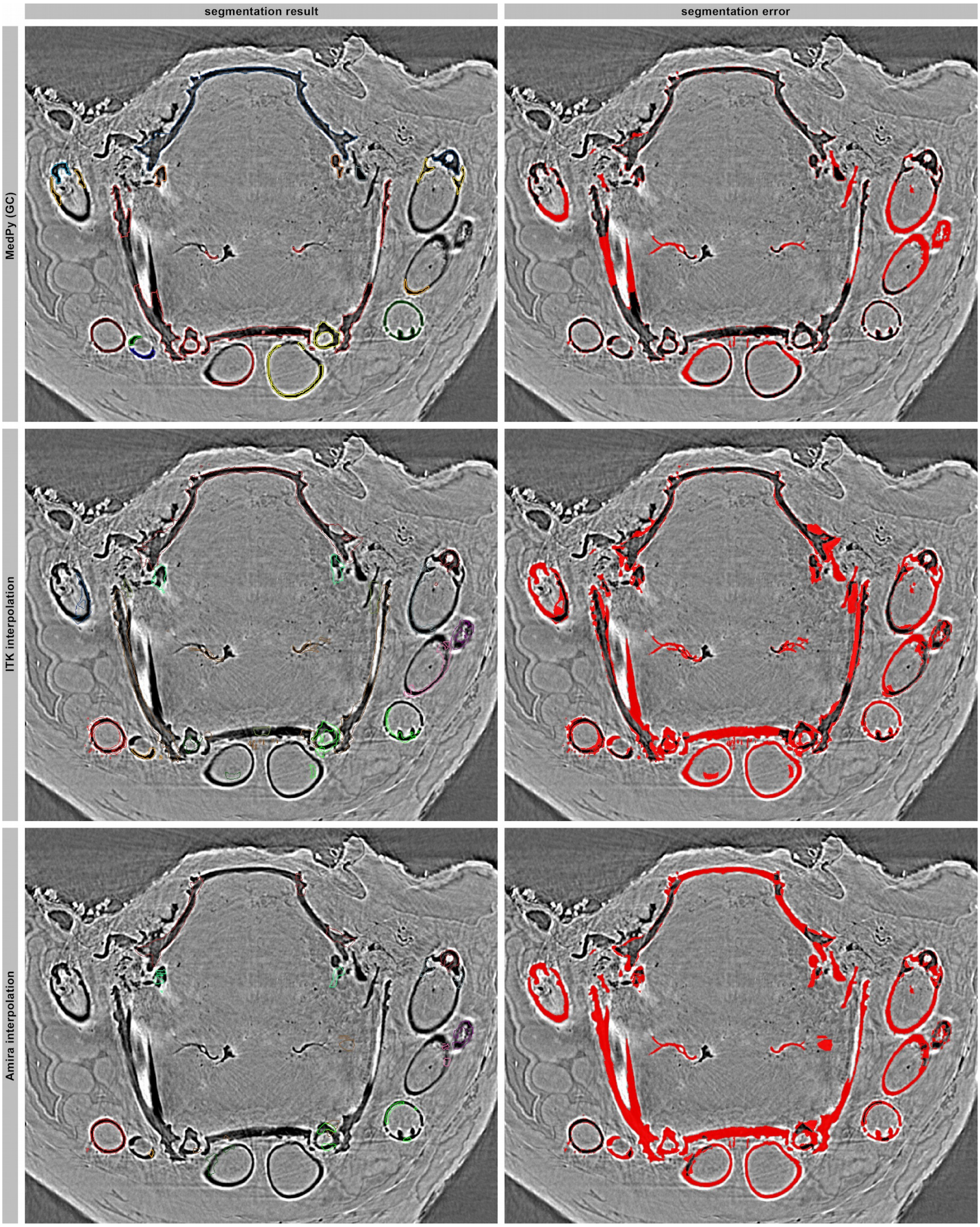

Supplementary Fig. 3 | Visual comparison of the segmentation results of different semi-automatic segmentation tools when segmenting a mineralized wasp. The standard configuration is used for all techniques. Half of the originally pre-segmented slices were used to obtain the segmentation results. The segmentation errors shown here are based on the remaining pre-segmented slices that have been labeled by an expert.

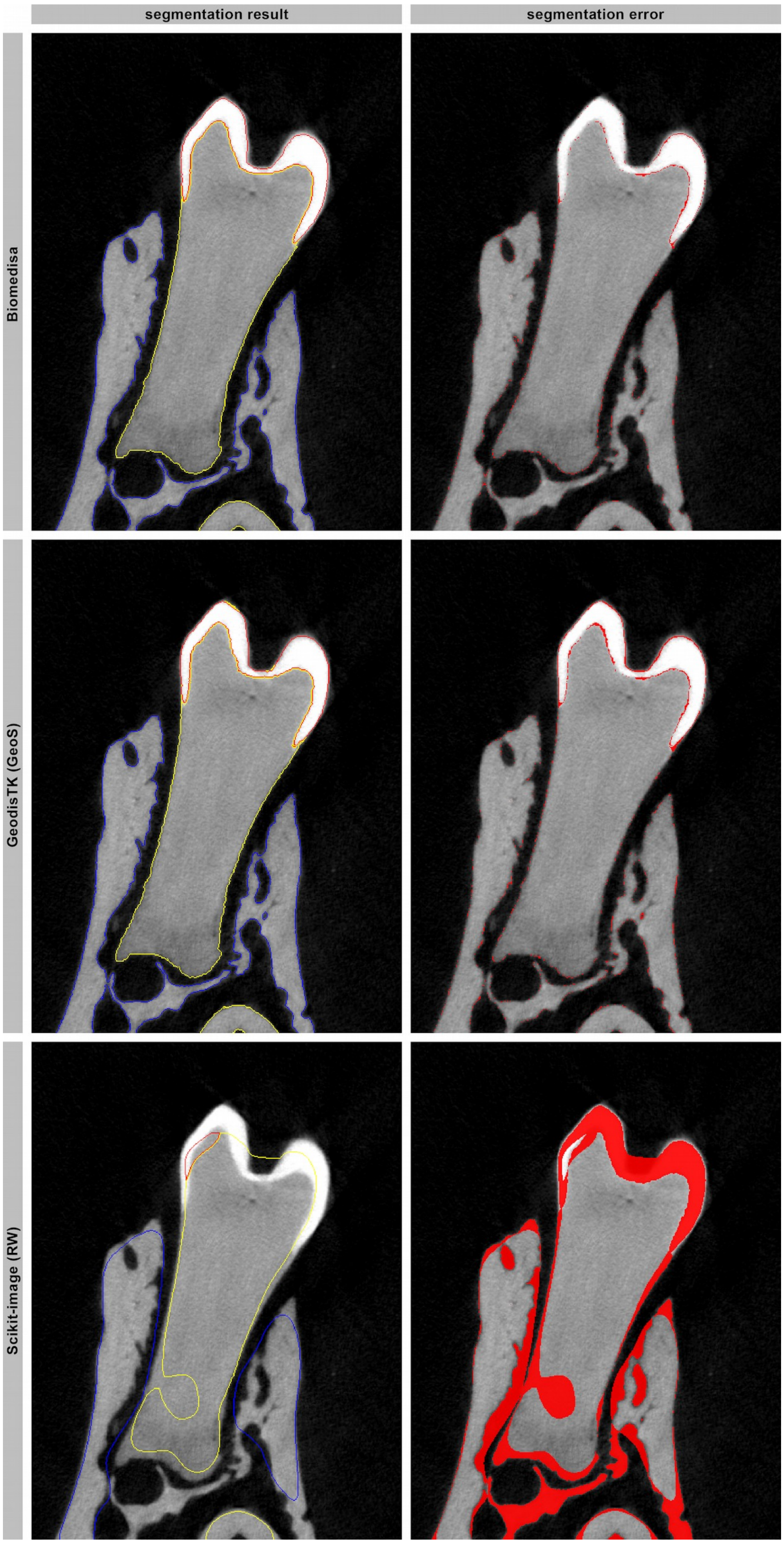

Supplementary Fig. 4 | Visual comparison of the segmentation results of different semi-automatic segmentation tools when segmenting a mouse molar. The standard configuration is used for all techniques. Half of the originally pre-segmented slices were used to obtain the segmentation results. The segmentation errors shown here are based on the remaining pre-segmented slices that have been labeled by an expert.

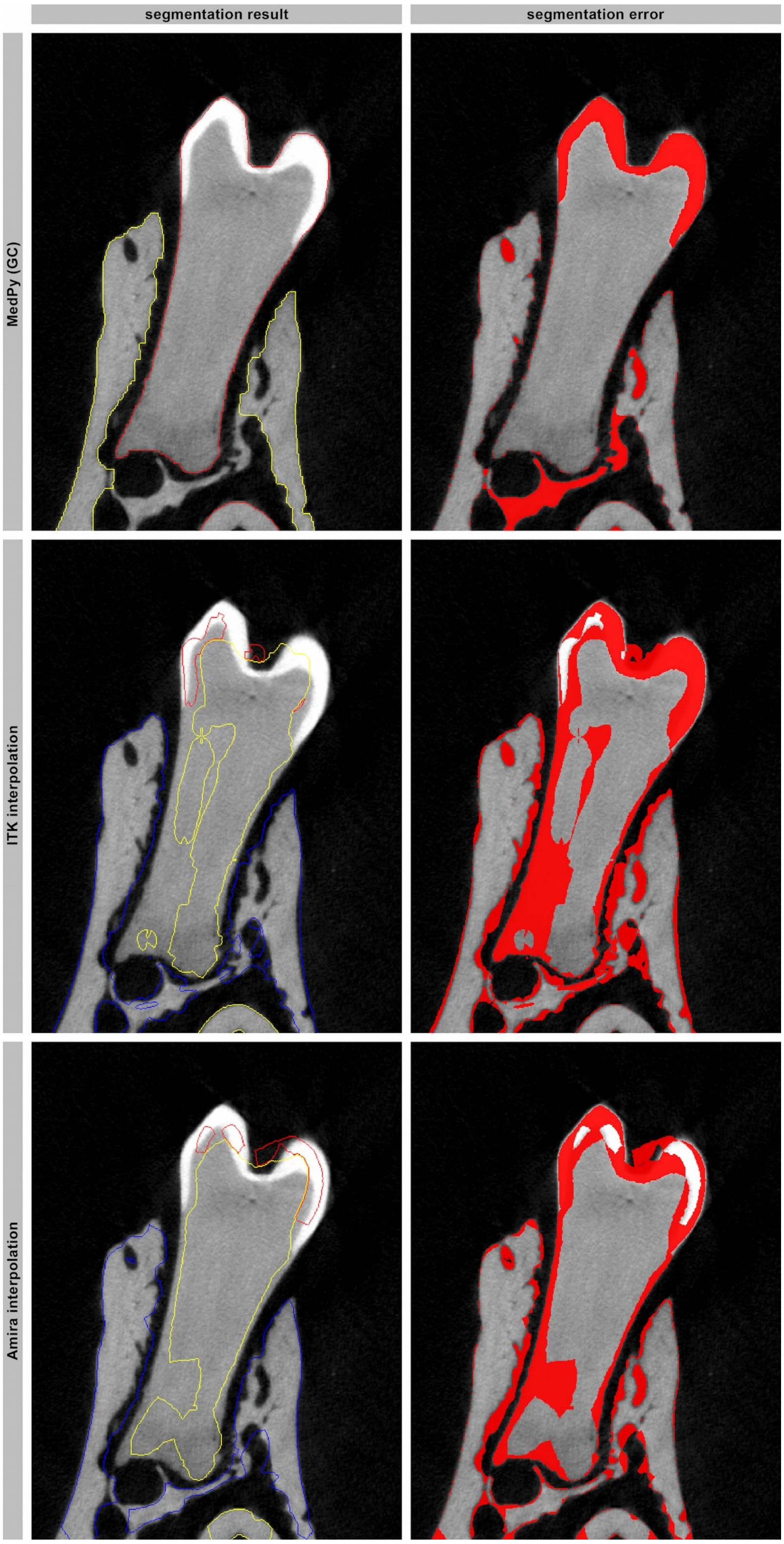

Supplementary Fig. 5 | Visual comparison of the segmentation results of different semi-automatic segmentation tools when segmenting a mouse molar. The standard configuration is used for all techniques. Half of the originally pre-segmented slices were used to obtain the segmentation results. The segmentation errors shown here are based on the remaining pre-segmented slices that have been labeled by an expert.

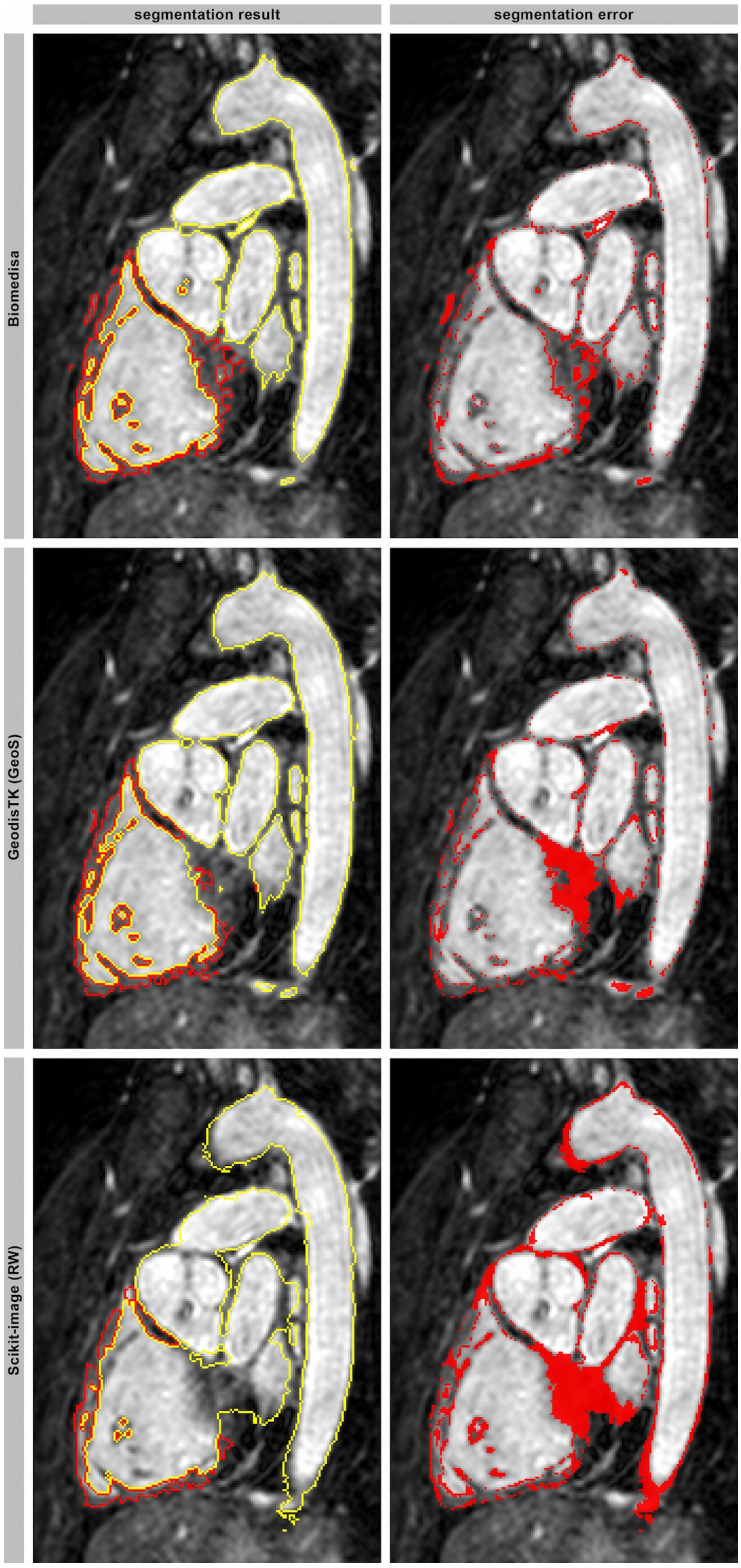

Supplementary Fig. 6 | Visual comparison of the segmentation results of different semi-automatic segmentation tools when segmenting a human heart. The standard configuration is used for all techniques. Every 20th slice of the ground truth data was used to initialize the algorithms and obtain the segmentation results. The segmentation errors shown here are based on the ground truth data that have been labeled by an expert.

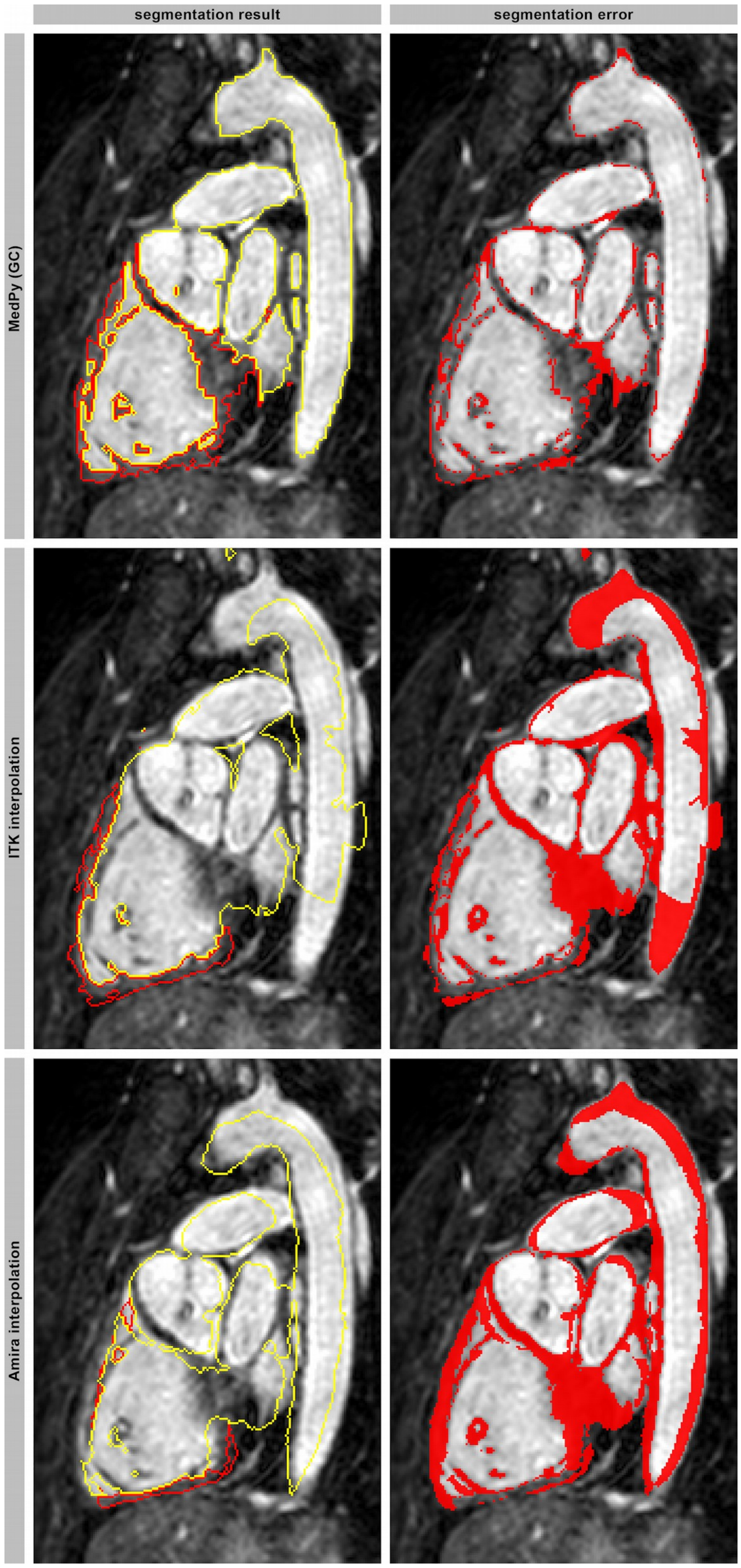

Supplementary Fig. 7 | Visual comparison of the segmentation results of different semi-automatic segmentation tools when segmenting a human heart. The standard configuration is used for all techniques. Every 20th slice of the ground truth data was used to initialize the algorithms and obtain the segmentation results. The segmentation errors shown here are based on the ground truth data that have been labeled by an expert.

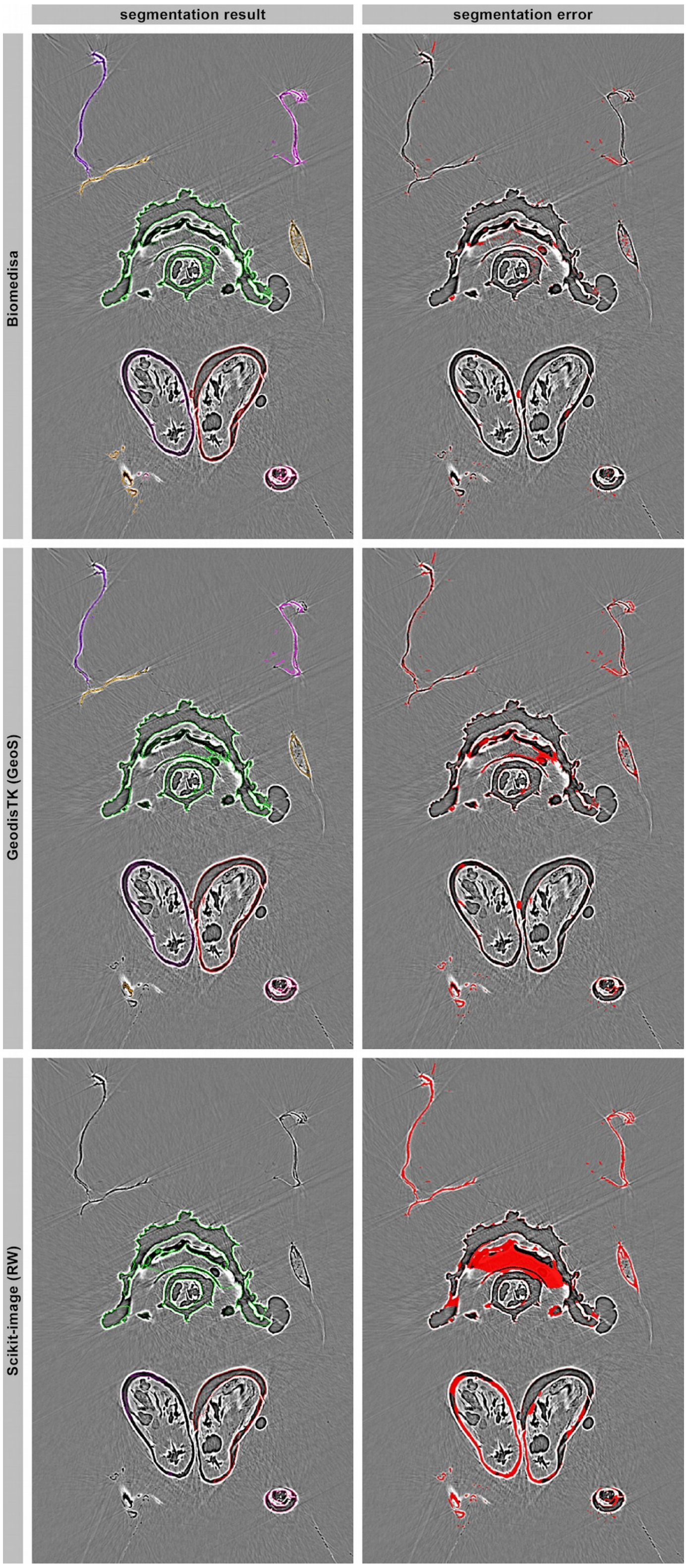

Supplementary Fig. 8 | Visual comparison of the segmentation results of different semi-automatic segmentation tools when segmenting a wasp from amber. The standard configuration is used for all techniques. Half of the originally pre-segmented slices were used to obtain the segmentation results. The segmentation errors shown here are based on the remaining pre-segmented slices that have been labeled by an expert.

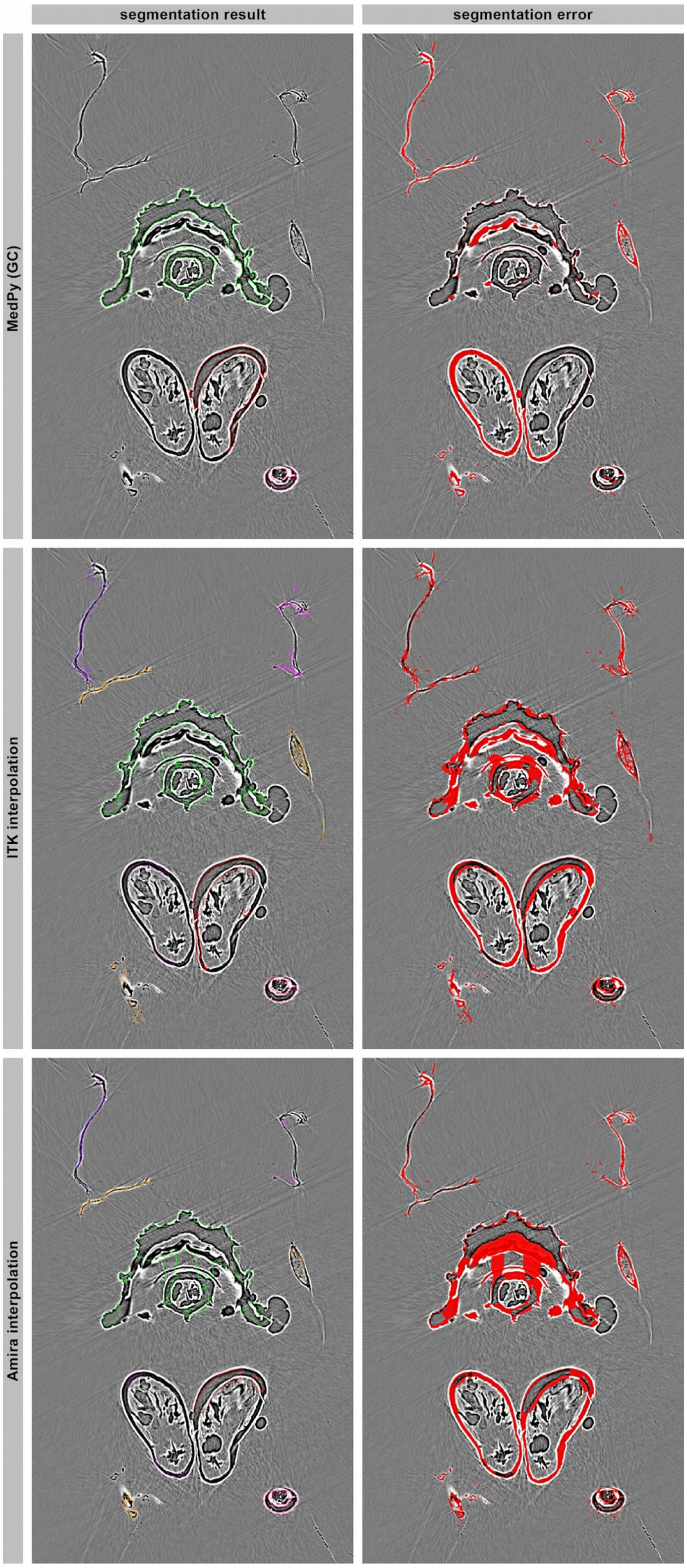

Supplementary Fig. 9 | Visual comparison of the segmentation results of different semi-automatic segmentation tools when segmenting a wasp from amber. The standard configuration is used for all techniques. Half of the originally pre-segmented slices were used to obtain the segmentation results. The segmentation errors shown here are based on the remaining pre-segmented slices that have been labeled by an expert.

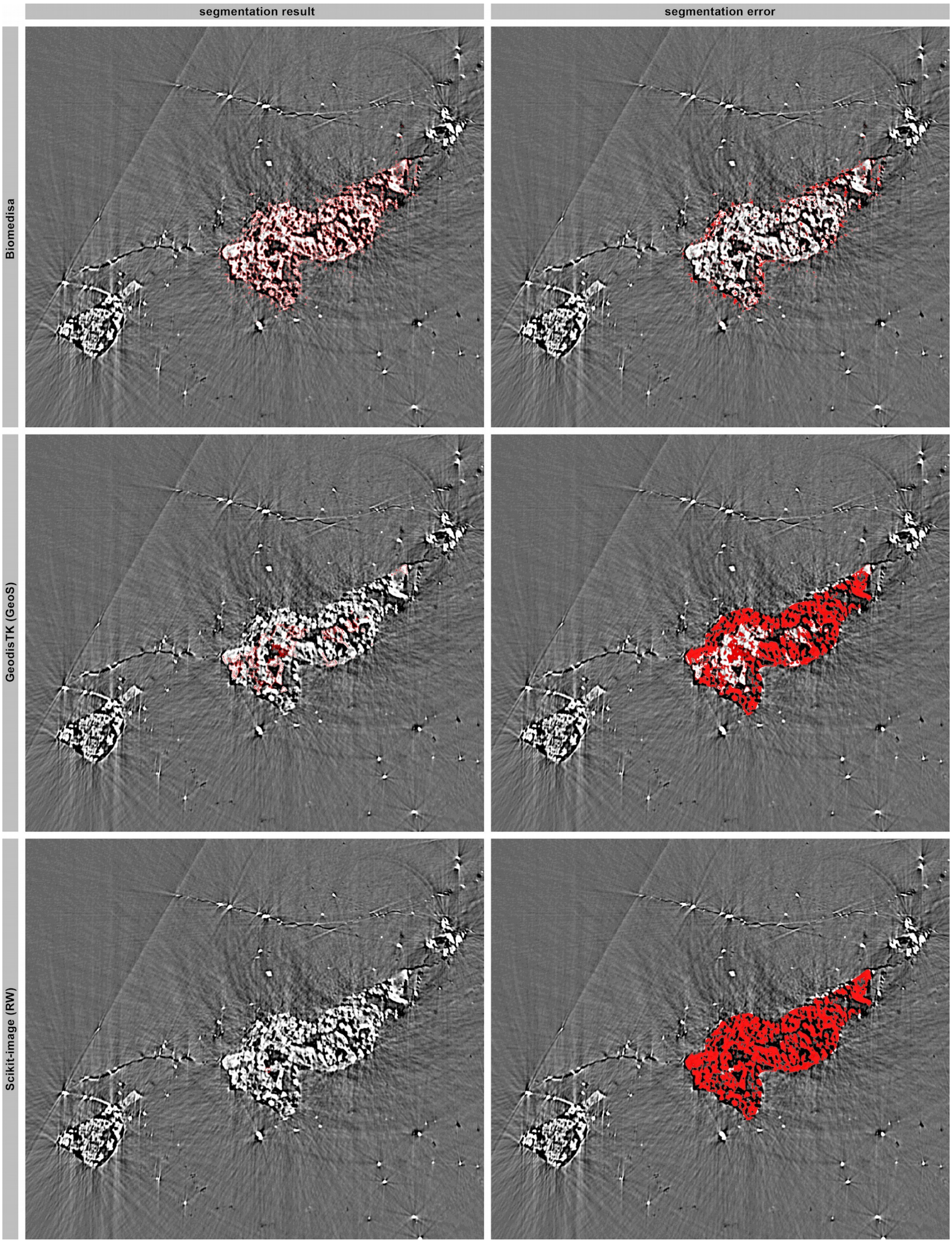

Supplementary Fig. 10 | Visual comparison of the segmentation results of different semi-automatic segmentation tools when segmenting a theropod claw. The standard configuration is used for all techniques. Half of the originally pre-segmented slices were used to obtain the segmentation results. The segmentation errors shown here are based on the remaining pre-segmented slices that have been labeled by an expert.

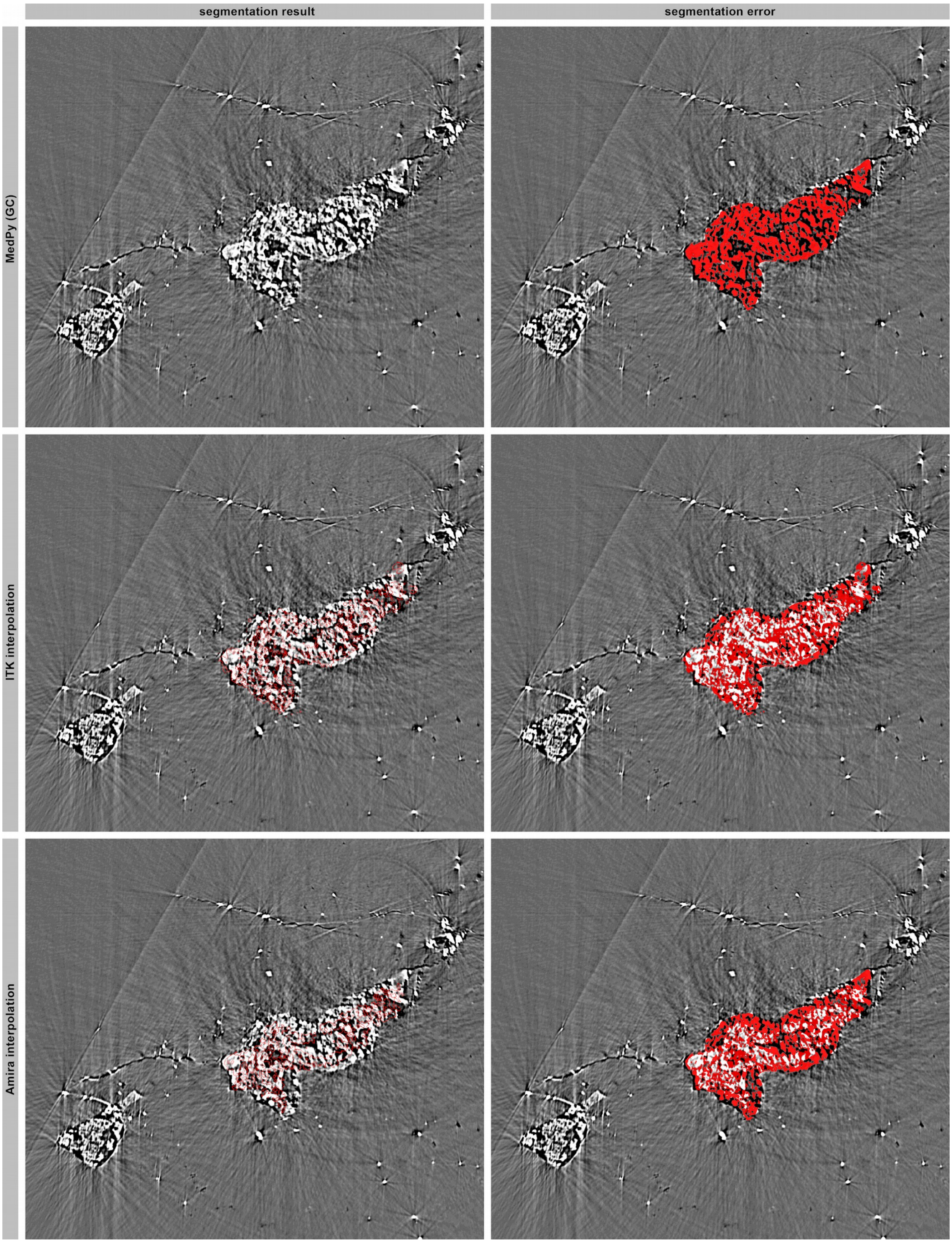

Supplementary Fig. 11 | Visual comparison of the segmentation results of different semi-automatic segmentation tools when segmenting a theropod claw. The standard configuration is used for all techniques. Half of the originally pre-segmented slices were used to obtain the segmentation results. The segmentation errors shown here are based on the remaining pre-segmented slices that have been labeled by an expert.

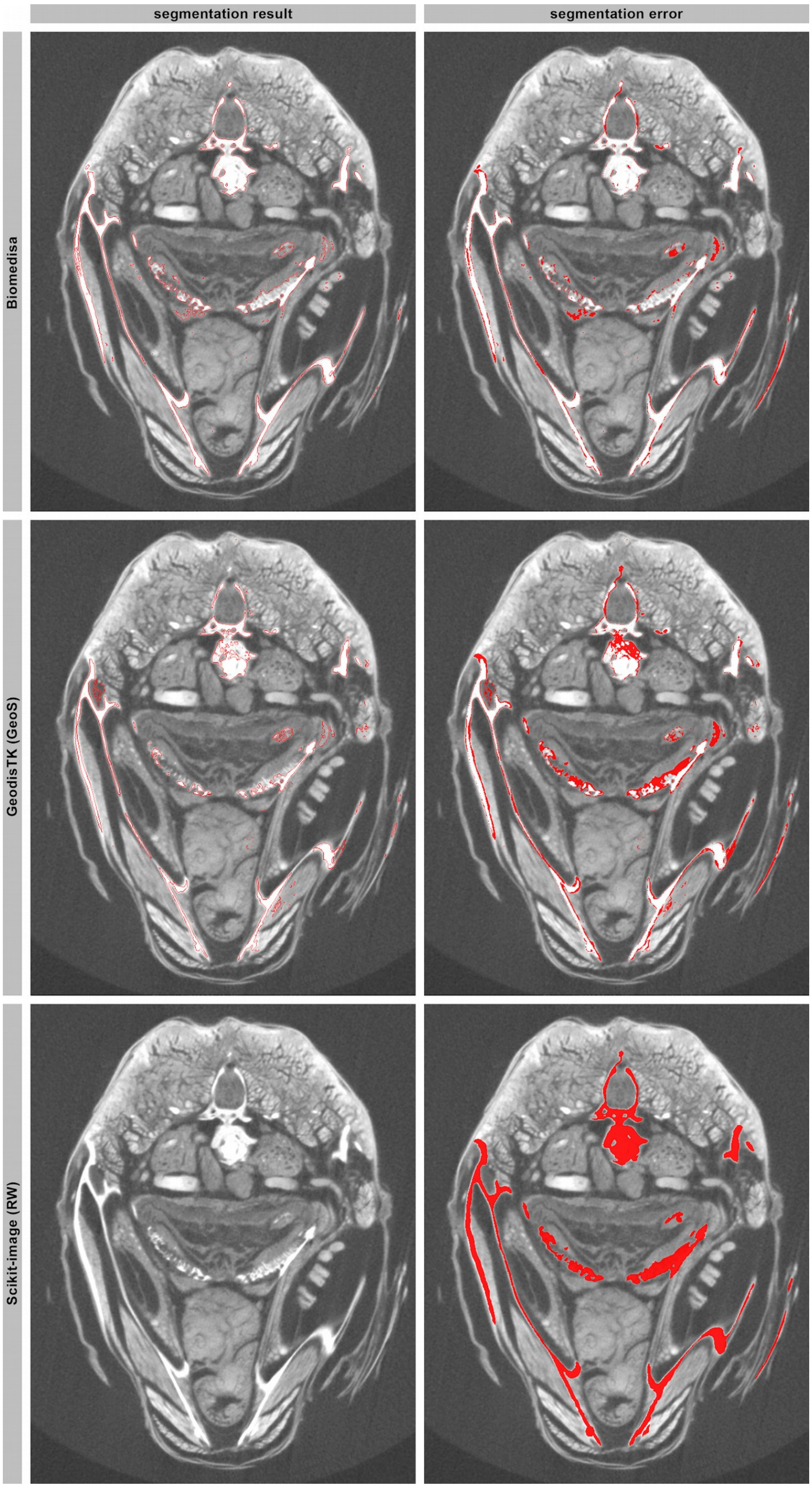

Supplementary Fig. 12 | Visual comparison of the segmentation results of different semi-automatic segmentation tools when segmenting the skeleton of a medaka fish. The standard configuration is used for all techniques. Half of the originally pre-segmented slices were used to obtain the segmentation results. The segmentation errors shown here are based on the remaining pre-segmented slices that have been labeled by an expert.

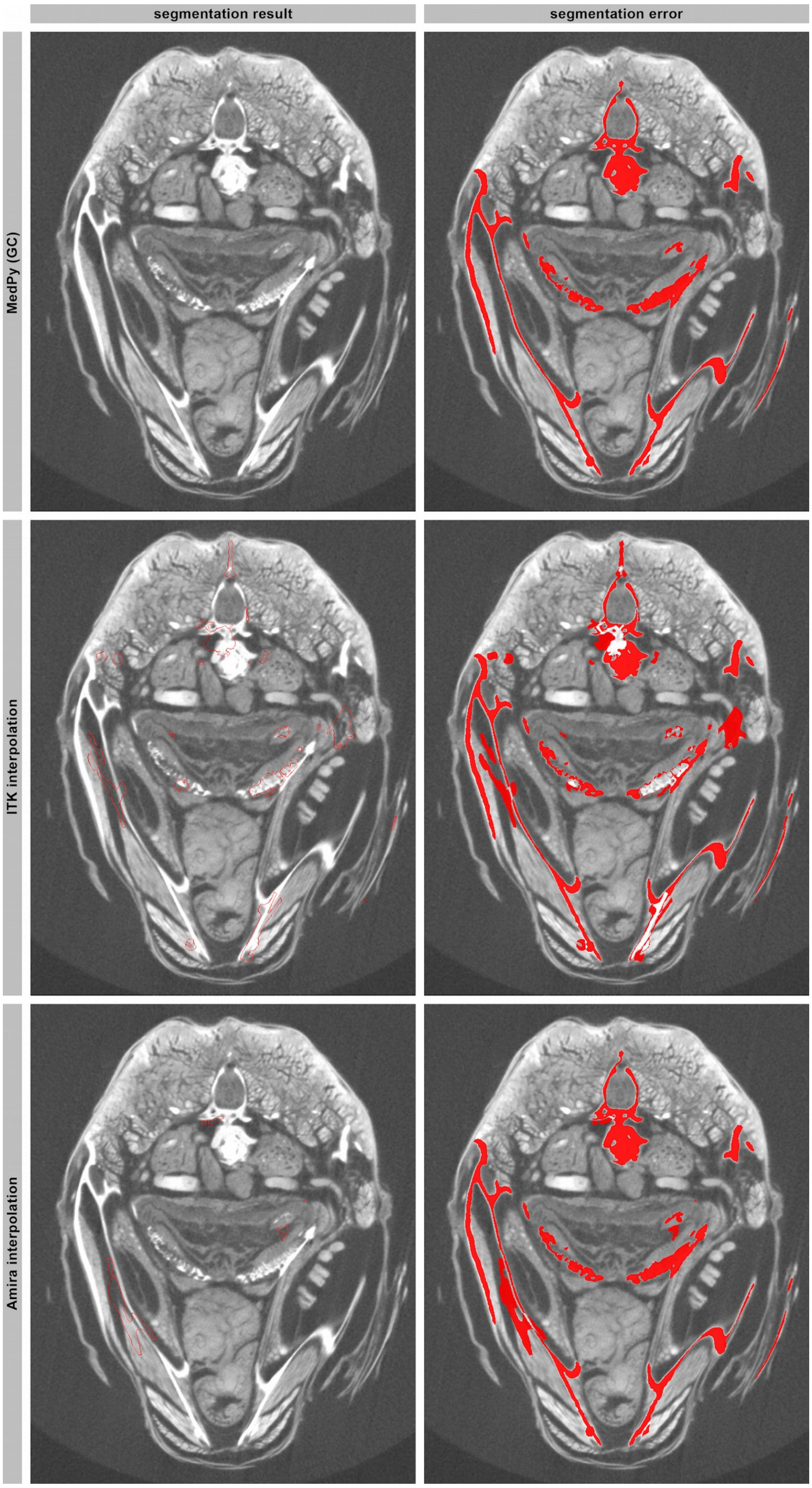

Supplementary Fig. 13 | Visual comparison of the segmentation results of different semi-automatic segmentation tools when segmenting the skeleton of a medaka fish. The standard configuration is used for all techniques. Half of the originally pre-segmented slices were used to obtain the segmentation results. The segmentation errors shown here are based on the remaining pre-segmented slices that have been labeled by an expert.

Supplementary Table 1 | Quantitative comparison of different semi-automatic segmentation tools for the segmentation of the *Trigonopterus* dataset. For the configuration, the values of the default parameters were chosen, i.e. β = 130 (RW), norw = 10 and sorw = 4000 (Biomedisa). Graph Cut and GeoS have no default values for σ and the number of iterations, respectively. The values were therefore chosen from the examples in the documentation, i.e. σ = 15 (GC) and iterations = 4 (GeoS). Dice scores were evaluated based on the manual segmentation of every 5th slice made by an expert. Highest accuracy and best result are shown in bold font. For comparison with a lower number of labels, the original 64 labels were merged into 11 related labels.

		Every 20th slice pre-segmented		Every 40th slice pre-segmented		Every 80th slice pre-segmented	
**Dataset**	**Method**	**Dice (%)**	**Time (min)**	**Dice (%)**	**Time (min)**	**Dice (%)**	**Time (min)**
Trigonopterus (64 labels, 1,497×734×1,117 voxels)	Biomedisa	**98.00**	25	**97.28**	15	**93.10**	10
	GeodisTK (GeoS)	97.50	402	95.91	433	91.57	462
	Scikit-image (RW)	91.64	388	79.44	507	45.44	648
	MedPy (GC)	58.99	2548	0.0	2584	0.0	2611
	ITK interpolation	86.86	13	78.91	10	62.25	6
	Amira interpolation	87.94	14	79.24	16	62.70	14
Trigonopterus (11 merged labels, 1,497×734×1,117 voxels)	Biomedisa	**98.13**	24	**97.47**	14	**93.53**	8
	GeodisTK (GeoS)	97.66	143	96.14	144	92.26	153
	Scikit-image (RW)	91.82	206	79.74	244	45.83	299
	MedPy (GC)	60.60	742	0.0	720	0.0	762
	ITK interpolation	86.29	17	78.21	14	58.55	11
	Amira interpolation	85.60	4	75.52	3	54.10	3

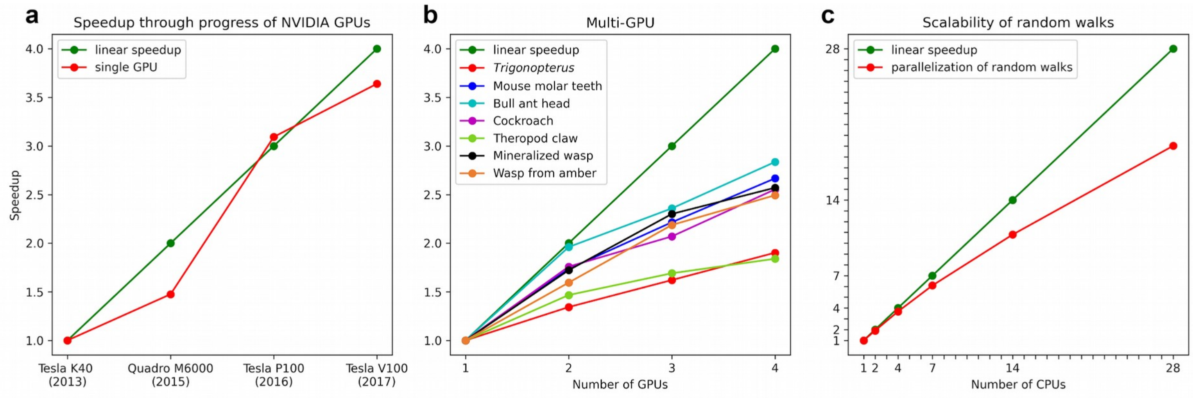

Supplementary Fig. 14 | Scalability of computing performance. a Speedup of the Biomedisa segmentation of the *Trigonopterus* dataset using a single GPU achieved through progress of NVIDIA GPU technology. **b** Speedup of the segmentation of different datasets using 1 to 4 NVIDIA Tesla V100 GPUs. **c** Speedup of calculating random walks gained when segmenting a mouse molar tooth using up to 28 CPUs.

Supplementary Table 2 | μCT datasets acquired by the authors

sample	medium	X-ray source	detector system	No. of projections	angular range	exposure time/frame	effective voxel size	reconstructed raw volume (voxels)	realigned and cropped volume (voxels)
Trigonopterus weevil	100% ethanol	Synchrotron bending magnet, peak at 15 keV, 0.2 mm Al filter	pco.dimax,10 μm LSO:Tb scintillator	3,000	180°	14.2 ms	2.44 μm	2,016 x 2,016 x 2,016	1,497 x 733 x 1,117
wasp in amber	Baltic amber	Synchrotron bending magnet, peak at 15 keV, 0.2 mm Al filter	pco.dimax, LuAG:Ce scintillator	3,000	180°	14.2 ms	1.22 μm	2,016 x 2,016 x 2,016 (3x)	1,417 x 2,063 x 2,733
mineralized wasp	apatite	Synchrotron bending magnet, peak at 15 keV, 0.2 mm Al filter	pco.dimax, 12 μm LSO:Tb scintllator	3,000	180°	14.2 ms	1.22 μm	2,016 x 2,016 x 2,016 (2x)	1,077 x 992 x 2,553
Bull ant queen	100% ethanol	Synchrotron bending magnet, peak at 25 keV, 1 mm Al & 50 μm Cu filter	pco.edge, 100 μm LuAG:Ce scintillator	3,000	180°	0.2 s	7.33 μm	2,560 x 2,560 x 2,150	1,957 x 1,165 x 2,321
theropod claw	Burmese amber	Synchrotron bending magnet, peak at 15 keV, 0.2 mm Al filter	pco.dimax, LuAG:Ce scintillator	3,000	180°	14.2 ms	1.22 μm	2,016 x 2,016 x 2,016 (2x)	1,986 x 1,986 x 3,602
cockroach	100% ethanol	X-ray tube, 80kV acceleration voltage, tungsten target, 20W target power	XRD1621 CN 14 ES, DRZ+ scintillator	2,048	360°	1.25 s	27.9 μm	2,048 x 2,048 x 1,900	613 x 606 x 1,927
medaka	agarose	X-ray tube, 90kV acceleration voltage, tungsten target, 10W target power	XRD1621 CN 14 ES, DRZ+ scintillator	2,048	360°	5 s	7.5 μm	2,048 x 2,048 x 1,900 (3x)	900 x 1,303 x 4,327
