# Supplementary material for: Introducing Biomedisa as an open-source online platform for biomedical image segmentation
Source: Nat Commun. 2020 Nov 4;11:5577. doi: 10.1038/s41467-020-19303-w (PMC7642381; doi:10.1038/s41467-020-19303-w)
Supplement: Supplementary file 2 — Reporting Summary [file 41467_2020_19303_MOESM2_ESM.pdf]

Corresponding author(s): Philipp D. Lösel

Last updated by author(s): Sep 24, 2020

Reporting Summary

Nature Research wishes to improve the reproducibility of the work that we publish. This form provides structure for consistency and transparency in reporting. For further information on Nature Research policies, see our Editorial Policies and the Editorial Policy Checklist.

Statistics

For all statistical analyses, confirm that the following items are present in the figure legend, table legend, main text, or Methods section.

n/a	Confirmed	
☒	☐	The exact sample size (*n*) for each experimental group/condition, given as a discrete number and unit of measurement
☒	☐	A statement on whether measurements were taken from distinct samples or whether the same sample was measured repeatedly
☒	☐	The statistical test(s) used AND whether they are one- or two-sided<br>*Only common tests should be described solely by name; describe more complex techniques in the Methods section.*
☒	☐	A description of all covariates tested
☒	☐	A description of any assumptions or corrections, such as tests of normality and adjustment for multiple comparisons
☒	☐	A full description of the statistical parameters including central tendency (e.g. means) or other basic estimates (e.g. regression coefficient) AND variation (e.g. standard deviation) or associated estimates of uncertainty (e.g. confidence intervals)
☒	☐	For null hypothesis testing, the test statistic (e.g. F, t, r) with confidence intervals, effect sizes, degrees of freedom and P value noted<br>*Give P values as exact values whenever suitable.*
☒	☐	For Bayesian analysis, information on the choice of priors and Markov chain Monte Carlo settings
☒	☐	For hierarchical and complex designs, identification of the appropriate level for tests and full reporting of outcomes
☒	☐	Estimates of effect sizes (e.g. Cohen's d, Pearson's r), indicating how they were calculated

Our web collection on statistics for biologists contains articles on many of the points above.

Software and code

Policy information about availability of computer code

Data collection	We used the UFO 0.16 framework (https://https://github.com/ufo-kit/ufo-core) for tomographic reconstruction.
Data analysis	Data were analyzed using Amira 5.6 & 2019.2, CINEMA 4D R20, Biomedisa 20.08.1 (https://biomedisa.org, https://github.com/biomedisa/biomedisa), GeodisTK 0.1.5.3, MedPy 0.4.0, scikit-image 0.15.0, ITK 5.1.0, TensorFlow 1.15.0, Keras 2.3.1, CUDA 10.2, PyCUDA 2019.1.2, OpenMPI 2.1.1, PyOpenCL 2019.1.2.

For manuscripts utilizing custom algorithms or software that are central to the research but not yet described in published literature, software must be made available to editors and reviewers. We strongly encourage code deposition in a community repository (e.g. GitHub). See the Nature Research guidelines for submitting code & software for further information.

Data

Policy information about availability of data

All manuscripts must include a data availability statement. This statement should provide the following information, where applicable:

- Accession codes, unique identifiers, or web links for publicly available datasets
- A list of figures that have associated raw data
- A description of any restrictions on data availability

Datasets Trigonopterus (Figs 1-4), mouse molar teeth (Fig. 5b), wasp from amber (Fig. 5d), cockroach (Fig. 5e), theropod claw (Fig. 5f), mineralized wasp (Fig. 5g) and bull ant queen (Fig. 5h) are available at https://biomedisa.org/gallery. Dataset human hearts (Fig. 5c) is from the MICCAI Workshop on Whole-Heart and Great Vessel Segmentation from 3D Cardiovascular MRI in Congenital Heart Disease (HVSMR 2016). Information on how to obtain the data can be found at http://segchd.csail.mit.edu. Dataset human mandibles (Fig. 7) can be downloaded at https://doi.org/10.6084/m9.figshare.6167726.v5. Further data will be made available from the corresponding author upon reasonable request.

Field-specific reporting

Please select the one below that is the best fit for your research. If you are not sure, read the appropriate sections before making your selection.

☒ Life sciences ☐ Behavioural & social sciences ☐ Ecological, evolutionary & environmental sciences

For a reference copy of the document with all sections, see nature.com/documents/nr-reporting-summary-flat.pdf

Life sciences study design

All studies must disclose on these points even when the disclosure is negative.

Sample size	This paper presents and evaluates a software platform and an image segmentaion algorithm. It does not deal with experimental series of similar samples. Therefore, no sample size calculation was required. In order to demonstrate the broad potential of our method, we selected datasets from different application scenarios (i.e. medical data, fossils, alcohol-preserved specimens, vetebrates & arthopods).
Data exclusions	No data were excluded from the analyses.
Replication	The results can be reproduced using the data and source-code provided or the online platform. All experiments were carried out several times using the same hardware infrastructure with the published source-code to verify the reproducibility of the experimental results. All attempts at replication were successful.
Randomization	The study does not include experimental series but is based on individual specimens.
Blinding	This paper does not deal with experimental series of similar samples, which would require statistical analysis. Blinding was therefore not applicable here.

Reporting for specific materials, systems and methods

We require information from authors about some types of materials, experimental systems and methods used in many studies. Here, indicate whether each material, system or method listed is relevant to your study. If you are not sure if a list item applies to your research, read the appropriate section before selecting a response.

Materials & experimental systems

n/a	Involved in the study	
☒	☐	Antibodies
☒	☐	Eukaryotic cell lines
☐	☒	Palaeontology and archaeology
☐	☒	Animals and other organisms
☐	☒	Human research participants
☒	☐	Clinical data
☒	☐	Dual use research of concern

Methods

n/a	Involved in the study	
☒	☐	ChIP-seq
☒	☐	Flow cytometry
☒	☐	MRI-based neuroimaging

Palaeontology and Archaeology

Specimen provenance	The sample "theropod claw", which was scanned in the scope of this study, was acquired on 28 Sep 2016 at First Fossil Wood Shop (Khine Thit Sar), Shop 29, 1st Floor, Saysan Rd., Bahan Township, Myanmar. Export Permit: Registration No. 1646, Ministry of Co-Operatives, Naypyitaw, Myanmar.<br>The datasets "wasp from amber" and "mineralized wasp" were not acquired during this study, but originate from other works:<br>Wasp from amber: the dataset is featured in Mikó et al. 2018.<br>Mineralized wasp: the dataset is featured in van de Kamp et al. 2018.<br>The articles are referenced accordingly in our paper.
Specimen deposition	Theropod claw: The sample belongs to the Collection of Patrik Müller (Käshofen, Germany) and is available upon reasonable request.<br>Mineralized wasp: The specimen is stored at the Natural History Museum of Basel with collection number NMB F2875.<br>Wasp from amber: The specimen is stored at Senckenberg Deutsches Entomologisches Institut (Müncheberg, Germany) with collection number DEI-GISHym31819.
Dating methods	No new dates are provided

☐ Tick this box to confirm that the raw and calibrated dates are available in the paper or in Supplementary Information.

Ethics oversight	No ethical approval or guidance was required. The theropod claw was legally acquired and exported and unaltered during the experiment. The paleontological samples "wasp from amber" and "mineralized wasp" were not scanned in the scope of this study. The respective tomographic datasets were provided by third parties (see above).

Note that full information on the approval of the study protocol must also be provided in the manuscript.

Animals and other organisms

Policy information about studies involving animals; ARRIVE guidelines recommended for reporting animal research

Laboratory animals	Two laboratory animals were scanned in order to obtain data used in the study (A Japanese rice fish (Medaka; Oryzias latipes): inbred iCab strain, male, 17 months old & a Dwarf hissing cockroach (Elliptorhina chopardi): male, ca. 10 months old). The dataset "mouse molar teeth" was not acquired during this study, but originates from a third party. The data is featured in Balanta-Melo et al. 2019 and was provided to use by the authors. The source is referenced in the article. The animals studied were adult BALB/c mice (Mus musculus; 9 weeks old).
Wild animals	The study does not involve wild animals.
Field-collected samples	The Trigonopterus weevil and the bull ant queen (Myrmecia pyriformis) were collected in the field and fixed in 100% ethanol. Both samples remained in ethanol during and after the tomographic scans.
Ethics oversight	The only vertebrate scanned for the study is the Japanese rice fish (Medaka). Animal husbandry and experimental procedures were performed at the Institute of Biological and Chemical Systems (IBCS) of Karlsruhe Institute of Technology (KIT) in accordance with German animal protection regulations (Regierungspräsidium Karlsruhe, Germany; Tierschutzgesetz 111, Abs. 1, Nr. 1, AZ35-9185.64/BH). The IBCS is under the supervision of the Regierungspräsidium Karlsruhe, who approved the experimental procedures.<br><br>All other animals involved are insects that do not require ethical approval or guidance.

Note that full information on the approval of the study protocol must also be provided in the manuscript.

Human research participants

Policy information about studies involving human research participants

Population characteristics	Not applicable as we only used data from third parties. See "Data selection" in Wallner et al. 2019 for human mandibles and http://segchd.csail.mit.edu/data.html for human heart datasets.
Recruitment	We did not recruit any human participants for this study. The datasets originate from third parties, which are referenced in the article.<br><br>Human mandibles: The datasets were collected during routine clinical practice in the Department of Oral and Maxillofacial Surgery at the Medical University of Graz in Austria (Wallner et al. 2019).<br><br>Human hearts: The data were provided by the organizers of the MICCAI Workshop on Whole-Heart and Great Vessel Segmentation from 3D Cardiovascular MRI in Congenital Heart Disease (HVSMR 2016, http://segchd.csail.mit.edu/) and featured in Pace et al. 2015.
Ethics oversight	To test our algorithm, we solely used data provided by third parties (see above). No human participants were involved in this study and thus no ethics oversight was required.

Note that full information on the approval of the study protocol must also be provided in the manuscript.
