# Supplementary material for: Introducing Biomedisa as an open-source online platform for biomedical image segmentation
Source: Nat Commun. 2020 Nov 4;11:5577. doi: 10.1038/s41467-020-19303-w (PMC7642381; doi:10.1038/s41467-020-19303-w)
Supplement: Supplementary file 6 — Description of Additional Supplementary Files [file 41467_2020_19303_MOESM6_ESM.pdf]

Title: Supplementary Movie 1
Description: Comparison between a conventional segmentation approach and Biomedisa using a Trigonopterus weevil as an example.

Title: Supplementary Movie 2
Description: Examples created with Biomedisa
